# P-1231. Optimal vancomycin AUC target in children with MRSA bacteremia: based on Bayesian-guided dosing

**DOI:** 10.1093/ofid/ofae631.1413

**Published:** 2025-01-29

**Authors:** Yonghee Lee, Gahee Kim, Jina Lee

**Affiliations:** Gangwon National University Hospital, Chuncheon, Kangwon-do, Republic of Korea; Asan Medical Center, University of Ulsan College of Medicine, Seoul, Kyonggi-do, Republic of Korea; Asan Medical Center, University of Ulsan College of Medicine, Seoul, Kyonggi-do, Republic of Korea

## Abstract

**Background:**

Despite the widespread use of vancomycin in methicillin-resistant *Staphylococcus aures* (MRSA) infections, there is still a lack of clinical data elucidating the optimal target concentration of vancomycin in the pediatric population.

**Methods:**

From September 2013 to December 2021, a retrospective analysis was conducted on pediatric patients (3 months- 18 years of age) with MRSA bacteremia. Vancomycin area under the concentration-time curve (AUC) was estimated by Bayesian software.

**Results:**

A total of 56 patients with MRSA bacteremia were included, with median age of 2.4 years. 92.9% had underlying medical conditions, most of which were associated with catheter-associated infections. Persistent bacteremia ≥ 48 hours, recurrent bacteremia, 30-day all-cause mortality, and acute kidney injury (AKI) were observed in 17.9%, 14.8%, 3.7%, and 7.1%, respectively. AUC_24-48_ ≥ 400 did not demonstrate clinical benefit in the above outcomes. Persistent bacteremia was associated with source control, 30-day all-cause mortality, and AKI. As an AUC target, AUC_24-48_ ≥ 528.2 mg·h/L was associated with persistent bacteremia (*P* < 0.01), and AUC_24-48_ ≥ 536.8 mg·h/L was associated with AKI (*P* = 0.01). No significant factors associated with 30-day mortality and recurrence were identified.

**Conclusion:**

We suggest an AUC_24-48_ < 528.2 mg·h/L as the optimal target for children aged ≥ 3 months with MRSA bacteremia. The lower bound of the target range was not identified.

**Disclosures:**

**All Authors**: No reported disclosures

